# Time impact on non-activated and kaolin-activated blood samples in thromboelastography

**DOI:** 10.1186/s12871-015-0033-9

**Published:** 2015-04-15

**Authors:** Miroslav Durila, Pavel Lukáš, Jiří Bronský, Karel Cvachovec

**Affiliations:** 1 Department of Anaesthesiology and Intensive Care Medicine, Second Faculty of Medicine, Charles University in Prague, University Hospital Motol, V Úvalu 84, 150 06, Prague, 5 Czech Republic; 2 Department of Paediatrics, Charles University, Second Faculty of Medicine, Prague, Czech Republic

**Keywords:** Citrated blood, Kaolin, Native, Non-activated, Thrombelastography

## Abstract

**Background:**

The correct methodology of thrombelastography might be influenced by elapsing time. In our study we investigated kaolin activated citrated samples together with non-activated citrated samples in relation to the elapsed times of 0, 15 and 30 minutes to compare both methods and to find out if there is an impact of time on results of thrombelastography.

**Methods:**

Blood samples obtained from 10 healthy volunteers were analyzed after 0, 15 and 30 minutes from sampling with kaolin activation and without activation. Then the results were analysed and compared between the non-activated and the kaolin-activated method.

**Results:**

All blood samples became more hypercoagulable with the time elapsing, both in non-activated and kaolin-activated samples and differences between both groups were found statistically and clinically significant after only 0 minutes.

**Conclusions:**

The non-activated citrated method seems to be reliable and suitable for thrombelastography in non-emergency cases (planned surgical procedures) when we have time to wait 15–30 minutes to get results. In urgent situations a rapid thrombelastography test should be preferred. Although the kaolin-activated method can also be used, results must be interpreted with caution.

## Background

Thrombelastography (TEG) has been used as a laboratory and bed-side method since 1948 [1]. However, it still has some inconveniences as do many of the methods being used when working with TEG, thus bringing a bit of chaos to the methodology and one of reasons to get faster results. Each hospital has its own method and that is why results cannot be compared among hospitals and experiences cannot be shared. Many methods have been introduced to practice such as the non-activated native and the kaolin-activated native method, as well as non-activated citrated and kaolin-activated citrated method and rapid TEG test. Other substances are also used to activate the sample, such as celite and tissue factor. Every method has advantages and disadvantages. The disadvantage of using the native method is the necessity to perform an analysis within 4–5 minutes from the sampling. This time period is very unpractical, especially during the admission of a patient in critical condition in the intensive care unit (ICU). Preparing a TEG machine requires our full attention and this time interval is often insufficient. A five minute interval is also insufficient to transport a sample from the operating theatre to the lab or to the place where a TEG is available. Therefore, the citrated method has been developed and is preferred by many authors [2-7]. Because the generation of TEG trace using non-activated citrated blood is slow, the addition of activators has been proposed to speed up the process. Kaolin is increasingly used for this purpose [2,8,9]. However, kaolin not only initiates the intrinsic coagulation pathway but has complex effects on platelets. It activates platelets by releasing platelet factor 3 [10] and can also inflict the clot to retract and mimic the fibrinolysis pattern [11] and so mislead doctors to the incorrect therapy. On the other hand, citrated samples are not stable over time and results are more hypercoagulable with the time elapsing [12]. Thromboelastography users might take advantage of the time effect in non-activated samples (without kaolin) to produce more cost-effective and consistent TEG results in non-emergency situations such as planned surgical interventions. This could be useful to reduce fresh frozen plasma administering (FFP) in patients with prolonged prothrombin time.

We decided to compare the results of kaolin activated citrated samples with results of non-activated sample in relation with elapsed time of 0, 15 and 30 minutes. We wanted to find out if time impacts the activation of citrated samples. The time period of 15 to 30 minutes would be very practical for transporting blood samples to lab. It is very important to elaborate the TEG method, which could be used in non-emergency situations (such as planned surgical procedures) especially in patients with intensive care unit (ICU) coagulopathy represented by prolonged prothrombin time (PT) because TEG as a global coagulation test may lead to reduced FFP transfusion administered preventively (before surgery) just on the base of prolonged PT and thus minimize related adverse side effects of FFP [13].

## Methods

This study was approved by the Ethics Committee for Multi-Centric Clinical Trials of the University Hospital Motol (date of approval March 2008, Reference No. EK425/08) and written informed consent was obtained from all subjects.

In the first step of study we investigated the role of time and its influence on the stability of blood samples after 0, 15 and 30 minutes in both non-activated citrated and kaolin activated citrated samples. Blood was obtained from 10 healthy volunteers (5 men and 5 women, from 20 to 30 years, non-smokers, not taking contraceptive pills, no bleeding disorder history) from the cubital vein using a 10 ml syringe and needle and blood was squirted into 3 citrated tubes with a volume of 2 ml (Vacuette, 9 NC Coagulation Sodium Citrate 3.2%, Greiner Bio-One GmbH, Austria). Immediately after obtaining blood (and gentle mixing the blood sample 5–6 times) - after 0 minutes, 1 ml of blood was poured from the first 2 ml citrated tube to the kaolin vial and after 3–4 minutes 340 μl of this blood was pipetted to the cuvette containing 20 μl of calcium (0.2 M CaCl_2_, TEG Hemostasis System, Haemoscope Corp., USA) and the sample was started. Meanwhile (after 2–3 minutes from sampling) 340 μl of blood was pipetted from the citrated tube to the other cuvette containing 20 μl of calcium and the non-activated sample was initiated. After 15 minutes the second 2 ml citrated tube was used and the process was repeated. After 30 minutes the third 2 ml citrated tube was used and the process was repeated again. Three TEG machines were used simultaneously in this study (6 channels used alternately, at 37°C, TEG Haemoscope, Niles, Illinois, USA).

In the second step we compared the results between non-activated and kaolin-activated method after 0, 15 and 30 minutes.

### Statistical analysis

GraphPad Prism was the statistics program used in our study. After data testing for normality using the D’Agostino & Pearson omnibus normality test, a repeated measures ANOVA (analysis of variance) was used to compare the effect of time on sample stability. Then in post hoc analysis the Tukey test was used. In the second step to compare non-activated with kaolin-activated, a paired t-test was used (p < 0.05 was considered significant).

## Results

**Figure 1:**
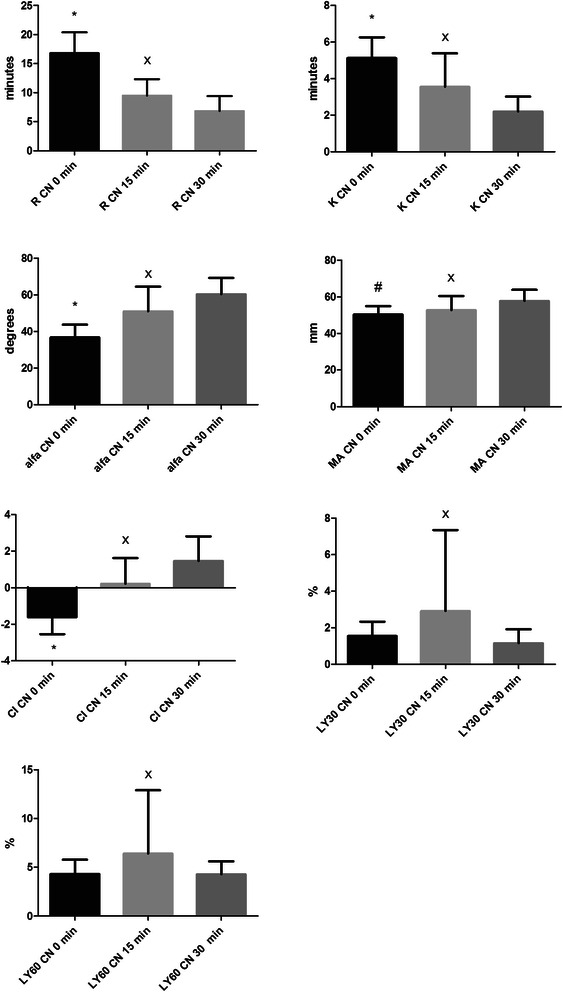
Differences of parameters in citrated non-activated (CN) group among 0, 15, 30 minutes. Little sign means statistical differences and error bars represent SD. R – reaction time, time from the start of the sample run to the first detectable clot formation (amplitude =2 mm); K – time from R to the clot amplitude of 20 mm (to specify the kinetics of the clot development); alfa angle, the angle formed by the slope between the amplitude of the trace at 2 mm and 20 mm; MA, maximum clot amplitude; CI, coagulation index; LY 30 and LY 60, level of fibrinolysis at 30 and 60 min, respectively, after MA was reached. * means significant differences between CN 0 min vs. CN 15 min and between CN 0 min vs. CN 30 min, p < 0.05; # means significant difference between CN 0 min vs. CN 30 min, p < 0.05; x means non-significant difference between CN 15 min vs. CN 30 min, p > 0.05.

**Figure 2:**
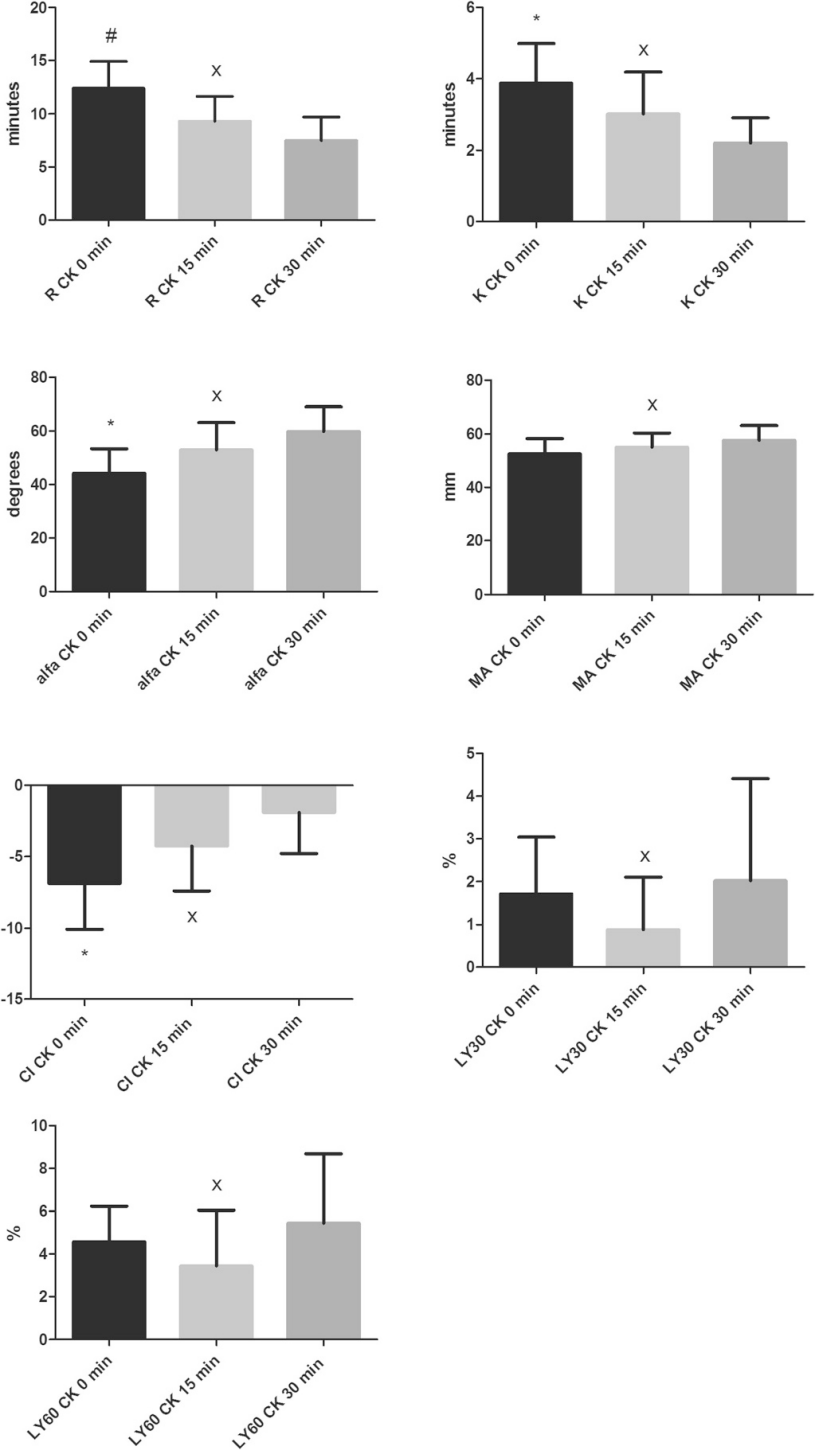
Differences of parameters in citrated kaolin-activated (CK) group among 0, 15, 30 minutes. Little sign means statistical differences and error bars represent SD. R – reaction time, time from the start of the sample run to the first detectable clot formation (amplitude =2 mm); K – time from R to the clot amplitude of 20 mm (to specify the kinetics of the clot development); alfa angle, angle formed by the slope between the amplitude of the trace at 2 mm and 20 mm; MA, maximum clot amplitude; CI, coagulation index; LY 30 and LY 60, level of fibrinolysis at 30 and 60 min, respectively, after MA was reached. * means significant differences between CK 0 min vs. CK 15 min and between CK 0 min vs. CK 30 min, p < 0.05; # means significant difference between CK 0 min vs. CK 30 min, p < 0.05; x means non-significant difference between CK 15 min vs. CK 30 min, p > 0.05.

In all measurements, both non-activated and kaolin activated, the time elapsed from blood sampling played a role and the samples became more hypercoagulable. The most significant time effect (p < 0.05) occurred within the first 15 minutes (between 0 and 15 minutes) both for non-activated and kaolin activated samples. After 15 minutes (between 15 and 30 minutes) time does not seem to play a significant role and the results do not differ significantly (p > 0.05) in either group (Figures 1 and 2). Interestingly, we found that CI in the non-activated method was nearest to 0 after 15 minutes whereas in kaolin-activated method was nearest to 0 after 30 minutes (Figures 1 and 2).

**Figure 3:**
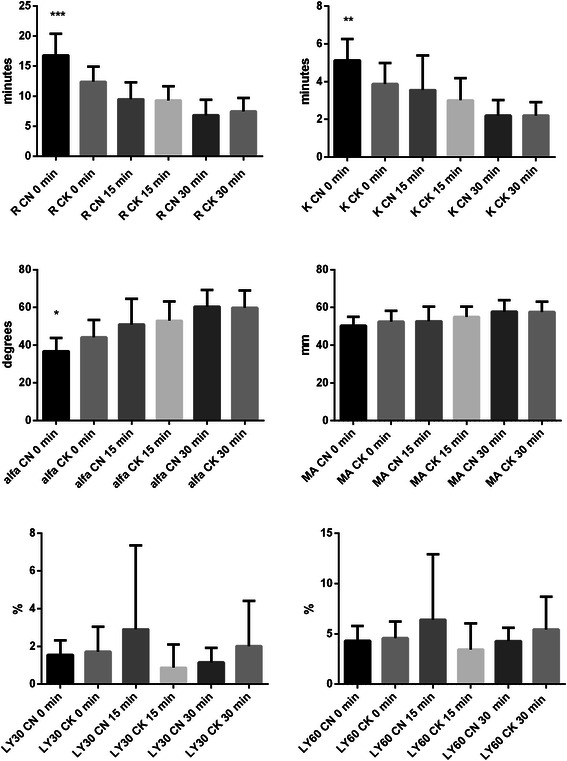
Differences between citrated non-activated (CN) and citrated kaolin-activated (CK) method for each parameter after 0, 15 and 30 minutes. Little sign means statically significant differences between methods within the same time group and error bars represent SD. R – reaction time, time from the start of sample run to the first detectable clot formation (amplitude =2 mm); K – time from R to the clot amplitude of 20 mm (to specify the kinetics of the clot development); alfa angle, angle formed by the slope between the amplitude of the trace at 2 mm and 20 mm; MA, maximum clot amplitude; LY 30 and LY 60, level of fibrinolysis at 30 and 60 min, respectively, after MA was reached. * means p < 0.05; ** means p < 0.01; *** means p < 0.001. Manufacturer references for citrated non-activated method: R 9–27 minutes, K 2–9 minutes, alfa angle 22–58 degrees, MA 44–64 mm, LY 30 0-8%, LY 60 0-15%. Manufacturer references for citrated kaolin-activated method: R 2–8 minutes, K 1–3 minutes, alfa angle 55–78 degrees, MA 51–69 mm, LY 30 0-8%, LY 60 0-15%.

In the second step we compared the results of non-activated samples with the results of kaolin-activated samples of all time points 0, 15, 30 minutes. Significant differences (p < 0.05) between non-activated and kaolin-activated samples were found mostly in 0 group for parameters R, K and alfa angle (Figure 3). After elapsing 15 and 30 minutes, there was no significant difference in TEG parameter (Figure 3) (p > 0.05).

## Discussion

Many studies have been done to investigate the time influence on sample stability, especially after 30 minutes to many hours elapsed from sampling, using the citrated method with either non-activated or kaolin activated samples [4,8,14,15]. In this study we investigated sample stability after 0, 15 and 30 minutes from sampling also in both activated and non-activated citrated samples. In practice this is the most interesting time because it is sufficient to transfer the sample to the laboratory and prevents redundant delay.

In both the non-activated and the kaolin-activated method, we found a tendency of hypercoagulability depending on the time elapsed from blood sampling. This is consistent with the findings of Camenzind who found that citrate does not completely inhibit thrombin formation [12]. The biggest impact of time effect was detected between 0 and 15 minutes in both methods (Figures 1 and 2). Afterwards there was no significant effect of time on coagulation. That means that blood samples can be analyzed between 15–30 minutes using both methods. This time is sufficient to transfer a blood sample from the patient to the lab where it can be analyzed. This laboratory usage of the TEG method may be sufficient and suitable for non-emergency cases where it is possible to wait some time for results, for example before planned surgical interventions (especially with ICU coagulopathy-prolonged PT). Thus it can help in decreasing the unnecessary administration of FFP which is usually administered preventively before surgery in case of prolonged PT. It is also recommended by other authors to wait at least 15 to 30 minutes to reach equilibration of the citrated blood sample before running TEG [12].

Our findings are also in accordance with results of White who did not find significant differences between results obtained after 15 and 30 minutes using the kaolin-activated citrated method [8]. Interestingly, our data obtained from healthy volunteers is very similar to his obtained from ICU patients who were even on anticoagulants and using arterial blood. The question remains, which time point is the best for analyses. Our volunteers are healthy and thus we believe that they do not have a tendency towards hypocoagulability or hypercoagulability and their CI is 0. As the CI in the non-activated method was nearest to 0 after 15 minutes, we believe that this time is the best for stabilization of the sample to get the most valid data. As the CI in kaolin-activated method was nearest to 0 after 30 minutes, this time period seems to be adequate for sample stabilization before analysis. However, as kaolin not only initiates the intrinsic coagulation pathway but as well activates platelets by releasing platelet factor 3 [10] and thus can inflict the clot to retract and mimic the fibrinolysis pattern [11], the non-activated method seems to be not only cheaper but also more reliable. The fibrinolysis TEG curve can be explained paradoxically by clot retraction which is accompanied by an increase of tensile strength. During this process formed fibrin filaments interacting with platelets are connecting the pin and cuvette. As soon as the retraction is so vehement that filaments start to break and disconnect the pin from the cuvette, it is detected by the TEG as a loss of resistance thus imitating fibrinolysis.

Another interesting finding of the study is that time and kaolin do not seem to have an effect on the parameters of fibrinolysis, although in one of our case reports we describe the effect of kaolin on the fibrinolysis pattern- in reality retraction of coagulum [11]. In the second step we investigated the relationship between results of non-activated citrated blood samples with results of kaolin-activated citrated blood samples of time points 0, 15, 30 minutes (groups analysed after 0, 15, 30 minutes from sampling). Surprisingly, significant differences were found mostly in the 0 time point group where the blood sample was analyzed immediately after sampling (after 0 minutes) (Figure 3). In the 15 and 30 minutes groups there were no differences in parameters. Therefore it could be rational to use time activation method in all cases when we have time to wait 15–30 minutes to get reliable results without using kaolin, thereby to get results not influenced by kaolin, which costs $5 US. This should also be confirmed in ICU patients and a new reference range for this time interval would also be reasonable.

The limitation of the study is that we analysed blood from healthy volunteers. Different situations may be observed in sick patients. Jabel et al. demonstrated that in ECMO patients citrated samples (kaolin activated) could produce an apparent heparin reversal effect [16]. This seems to be another reason why the native method should be prefered to get reliable results. On the other hand, Moreland et al. demonstrated artificial fibrinolysis when citrated samples were used in the heparinase TEG cups [17]. As well, we have published the observation that the heparinase method can mimic fibrinolysis even in native blood (not citrated blood), but in reality it is the hyperretraction of coagulum [18].

A second limitation that has to be mentioned is that the observed time effect found in healthy volunteers will need to be confirmed in ICU patients before assuming that the time effect would lead to equivalent results in this population.

The relatively small sample size of the study (10 volunteers) considering the relative large interindividual variability of TEG data is another limitation of the study. However differences were statistically significant.

## Conclusions

In conclusion, the time activation of the blood sample plays an important role in thrombelastography both in the kaolin activated and the non-activated method citrated method. Non-activated citrated TEG method used between 15–30 minutes from sampling seems to be reliable and suitable for analysing blood coagulation in haematology lab. This may be especially appropriate when TEG is used for whole blood coagulation assessment in patients with ICU coagulopathy (prolonged PT) before planned surgical intervention when FFP is administered preventively. This method of using TEG can help in decreasing often unnecessarily administered FFP.

### Ethical standards

Authors declare that the experiments comply with the current laws of Czech republic performed.
